# Some Scientific Aspects of Insanity

**Published:** 1891-10-24

**Authors:** 


### Some Scientific Aspects of Insanity.

### I.—BRAIN TISSUE.

When we see an individual for the first time, we look at the features, the shape of the limbs, and the entire bodily configuration, and we at once form a conception of his mental character and personality. In one sense such a view is the correct and only possible one, for it is found by experience that mental qualities are often reflected externally. In another and deeper sense it may with truth be asserted that such a conception is only superficial, and that the real being, the nervous system, is concealed from our gaze in much the same way as a crab or an oyster is hidden within its shell. The real living organisation of every human being is the nervous system. The outer visible parts of the body subserve two important purposes to this organism. First, they offer a protection or covering; and, second, they afford an accumulation of ministering instruments by means of which the nervous system becomes conscious of its surroundings, performs the various functions necessary for its health and continued existence, and moves about from place to place as the necessities of the individual demand. The nervous system, then, may be compared to such an animal as a crab or crayfish, provided with claws and feelers, and enclosed within a hard bony covering.

For the purpose of anatomical description it may be divided into three separate parts, all of which are more or less intimately associated in structure and function: (1) The brain; (2) the spinal cord; (3) the system of nerves.

1. The brain is entirely contained within the cavity of the skull. The skull in the adult is a hard, unyielding, bony box, and the brain substance, with its blood-vessels and membranes, occupies the entire cavity. Surrounding the whole brain are three membranes, one within the other. The outermost membrane, which lies next the skull, is thick and leathery, and is called the dura mater. The second membrane within the dura mater is called the arachnoid mater. The third membrane, contained within the arachnoid, is closely attached to the brain substance, and dips in between every little fold and fissure. It is called the pia mater.

It may be said that there are two brains within the cavity of the skull; the great brain, or cerebrum, which occupies by far the larger part of the cavity, and the little brain, or cerebellum, which occupies only a very small part of that cavity behind, below and almost concealed from view by the cerebrum. It will be sufficient here to state, with regard to the cerebellum, that its functions are believed to be exclusively concerned in the regulation of the various complex movements of the body by balancing and co-ordinating the same. But as these functions do not concern us in the study of insanity, the cerebellum will not again be referred to in these lectures.

The cerebrum is divided into two complete halves, a right and a left, which are united together in the middle and underneath by a broad white band, called the corpus callosum. These two divisions are called the right and left hemispheres of the brain. They resemble each other closely in every particular, so that in describing one we describe both. Each hemisphere is convex on its outer surface, and is pointed in front and behind. It is flattened on the inner surface where it is applied to its fellow. A rough approximation of the shape may be had by conceiving a lemon to be divided longitudinally into two equal halves. If we examine the surface of one of the hemispheres we find all over it little folds or ridges, called convolutions. There are also in certain positions on the surface deeper and longer lines than the ordinary divisions between the convolutions, and these lines map off the brain into divisions called lobes. There are four of these lobes. There are in front the frontal lobes, divided by the line called the fissure of Rolando, from the parietal lobe lying immediately behind it. This, again, is divided from the occipital lobe, which occupies the extreme posterior end of the hemisphere by a fissure called the parieto occipital fissure; while the fourth lobe, which lies below the others and projects forward, is mapped off from them by the great fissure of Sylvius. It is called the temporal lobe. In the living brain each of these lobes performs separate functions, which will be hereafter referred to.

We have now seen that the brain is divided into two symetrical equal halves but there is another division—the division of substance. If the brain is sliced with a sharp knife and one of the slices examined with the naked eye it will be found to be composed of two separate forms of matter called the gray matter and the white matter respectively. The gray matter forms the hind or outermost layer of the brain and dips into all the grooves between the convolutions; in short, the convolutions themselves are formed of gray matter. The white matter forms the centre of the brain and sends a projecting core into each convolution. Not only do these two kinds of matter differ in colour, they also differ in structure. We shall first take up the structure of the gray matter. When viewed under the microscope the gray matter is found to consist of innumerable multitudes of very minute pear-shaped cells. These cells are arranged in layers. The cells of one layer differ slightly from those of another layer in size and configuration, but the general principle of their construction is the same. They are minute little sacks from the sides of which numerous small processes or branches go to unite them with each other so that there is a continuity of action throughout the gray matter. Besides the branches from the sides of the cell a strong branch from the larger cells passes downwards into the white matter. The peculiar colour of the gray matter from which it derives its name is due to the large amount of blood which is required for the nourishment of its cells in the performance of the highest and most complex actions of which the individual is capable. These cells of the gray matter can best be compared to small electric batteries. It is in them that all the nervous force of the body is stored. It is by them that all the sensations from the outer world are felt and perceived, and it is from them that all the energy and nervous force for the movement and nutrition of the body emanates.

